# Characterization and Chemical Synthesis of Cm39 (α-KTx 4.8): A Scorpion Toxin That Inhibits Voltage-Gated K^+^ Channel K_V_1.2 and Small- and Intermediate-Conductance Ca^2+^-Activated K^+^ Channels K_Ca_2.2 and K_Ca_3.1

**DOI:** 10.3390/toxins15010041

**Published:** 2023-01-05

**Authors:** Muhammad Umair Naseem, Georgina Gurrola-Briones, Margarita R. Romero-Imbachi, Jesus Borrego, Edson Carcamo-Noriega, José Beltrán-Vidal, Fernando Z. Zamudio, Kashmala Shakeel, Lourival Domingos Possani, Gyorgy Panyi

**Affiliations:** 1 Department of Biophysics and Cell Biology, Faculty of Medicine, Research Center for Molecular Medicine, University of Debrecen, Egyetem ter. 1, 4032 Debrecen, Hungary; 2 Departamento de Medicina Molecular y Bioprocesos, Instituto de Biotecnologia, Universidad Nacional Autónoma de México, Av. Universidad 2001, Cuernavaca 62210, Morelos, Mexico; 3 Grupo de Investigaciones Herpetológicas y Toxinológicas, Centro de Investigaciones Biomédicas, Departamento de Biología, Facultad de Ciencias Naturales, Exactas y de la Educación, Universidad del Cauca, Sector Tulcan, Calle 2 N 3N-100, Popayán 190002, Cauca, Colombia

**Keywords:** Cm39, scorpion toxin, *Centruroides margaritatus*, K_V_1.2, K_Ca_2.2, K_Ca_3.1, electrophysiology, Ca^2+^-activated channel

## Abstract

A novel peptide, Cm39, was identified in the venom of the scorpion *Centruroides margaritatus*. Its primary structure was determined. It consists of 37 amino acid residues with a MW of 3980.2 Da. The full chemical synthesis and proper folding of Cm39 was obtained. Based on amino acid sequence alignment with different K^+^ channel inhibitor scorpion toxin (KTx) families and phylogenetic analysis, Cm39 belongs to the α-KTx 4 family and was registered with the systematic number of α-KTx 4.8. Synthetic Cm39 inhibits the voltage-gated K^+^ channel hK_V_1.2 with high affinity (K_d_ = 65 nM). The conductance–voltage relationship of K_V_1.2 was not altered in the presence of Cm39, and the analysis of the toxin binding kinetics was consistent with a bimolecular interaction between the peptide and the channel; therefore, the pore blocking mechanism is proposed for the toxin–channel interaction. Cm39 also inhibits the Ca^2+^-activated K_Ca_2.2 and K_Ca_3.1 channels, with K_d_ = 502 nM, and K_d_ = 58 nM, respectively. However, the peptide does not inhibit hK_V_1.1, hK_V_1.3, hK_V_1.4, hK_V_1.5, hK_V_1.6, hK_V_11.1, mK_Ca_1.1 K^+^ channels or the hNa_V_1.5 and hNa_V_1.4 Na^+^ channels at 1 μM concentrations. Understanding the unusual selectivity profile of Cm39 motivates further experiments to reveal novel interactions with the vestibule of toxin-sensitive channels.

## 1. Introduction

Potassium ion (K^+^) channels regulate several vital physiological processes such as membrane potential, cell volume control, calcium signaling, cell proliferation and action potential firing in both excitable and non-excitable cells [1,2]. Voltage-gated potassium (K_V_) channels constitute the largest family of K^+^ channels with other smaller families of K^+^ channels that are stimulated either by calcium ions or those that are constitutively active [3,4]. K_V_ channels are assembled by four homologous subunits, with each subunit consisting of six transmembrane segments (TM); four TM segments are part of the voltage sensing domain (VSD) and the other two TM segments form the pore domain [5]. Like K_V_ channels, small-conductance/intermediate-conductance Ca^2+^-activated K^+^ (K_Ca_) channels are also present in tetrameric form and each subunit has six TM segments; however, unlike K_V_ channels, K_Ca_ channels are insensitive to the changes in membrane potentials due to fewer positively charged amino acids in segments corresponding to the VSD [6,7]. Pharmacological modulation of K^+^ channels have therapeutic potential to treat autoimmune diseases, neuronal and cardiac disorders and cancers [8,9,10,11,12]. For example, deletion or mutation in *KCNA2* gene leads to the gain-of-function of K_V_1.2 channels, resulting in neuroexcitability disorders which can be treated using K_V_1.2 inhibitors [13,14]. Intermediate-conductance K_Ca_ (IK_Ca_ or K_Ca_3.1) and voltage-gated K_V_1.3 channels are the predominant K^+^ channels in human T lymphocytes which critically regulate the activation and proliferation processes [15]. The differential expression level of these K^+^ channels on T-cell subtypes (naïve and central memory T cells: K_Ca_3.1^high^ K_V_1.3^low^, effector memory T cells: K_Ca_3.1^low^ K_V_1.3^high^) allows specific manipulation of their activation using the selective inhibitors of K_V_1.3 or K_Ca_3.1, thereby achieving the targeted immunosuppressive outcomes [16,17]. Small conductance Ca^2+^-activated channels (K_Ca_2) are mainly expressed in neurons; because of their involvement in controlling the neuronal excitability and firing frequency, they have been proposed as potential targets for numerous neurological diseases [16]. Selective inhibition of K_Ca_2.2 with apamin (a peptide toxin from bee venom) may improve the cognitive performance in dementia and function as memory booster [18,19]. Therefore, selective inhibition of the K^+^ channels bears a huge potential to treat a variety of channelopathies.

A wide range of naturally occurring peptide toxins, especially from scorpion venom, target K^+^ channels. These peptides have played a crucial role in understanding the physiological functions of mammalian K^+^ channels and provided a tool to identify the therapeutic targets associated with these channels [20,21,22]. More than 198 scorpion toxins are known to block K^+^ channels with variable selectivity among different ion channels. Based on the cysteine scaffold, conserved amino acids and biological activity, potassium channel scorpion toxins (KTxs) have been classified into seven different families: α-KTx, β-KTx, γ-KTx, δ-KTx, ε-KTx, κ-KTx and λ-KTx [23]. α-KTxs are the most abundant toxins found in scorpion venom and contain 23–42 amino acids adopting a cystine-stabilized αβ fold with three to four disulfide bridges [24]. α-KTxs were further classified into 31 subfamilies according to their primary structure similarities (kaliumdb.org). A critically positioned lysine residue and an aromatic amino acid in the peptide toxin constitute a “functional dyad” which is generally considered a common feature of high-affinity K^+^ channel blocking peptides. The lysine residue protrudes deep into, and plugs, the pore of K_V_ channels [25,26].

*Centruroides margaritatus*, a scorpion belongs to the *Buthidae* family, is a synanthropic species whose venom is less toxic, with an LD_50_ of 59.9 mg/kg [27]. However, previous studies report that the intravenous dose of a chromatographic fraction (containing peptides between 2.5–6 kDa) of its venom caused severe toxic effects in rats as biological models [28]. A detailed characterization of a Colombian scorpion *C. margaritatus* venom was performed previously by our group [29] and we discovered two new K_V_ channel blocker peptide toxins: 1) CmERG1 (γ-KTx 10.1) that fully blocks the human *ether-à-gogo-Related* gene (hERG1) K^+^ channel (K_V_11.1) with high affinity, and 2) Cm28 (α-KTx 32.1) which is a high affinity inhibitor of K_V_1.2 and K_V_1.3 channels [30]. In the current work, while screening the venom components against various potassium ion channels, we identified another peptide toxin with a molecular weight of 3980 Da, named Cm39. Here we report the primary structure and chemical synthesis of Cm39 and the characterization of its pharmacological activity on ten different K^+^ channels and two Na^+^ channels by single-cell electrophysiology (patch-clamp) assay. Comparison of the amino acid sequence of Cm39 with other known KTxs and phylogenetic analysis showed great resemblance with α-KTx 4 members.

## 2. Results

### 2.1. Purification of Cm39 and Primary Sequence Determination

**Figure 1:**
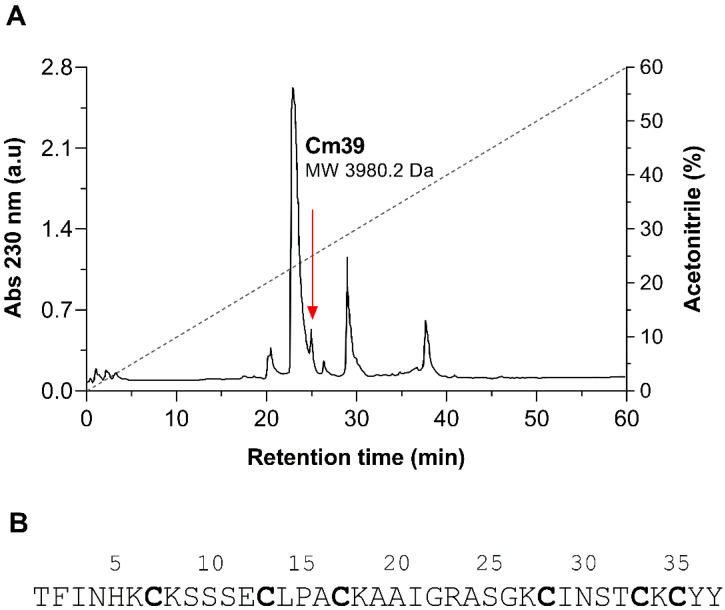
Isolation of native Cm39 from the venom of *C. margaritatus*. (**A**) Final step HPLC purification of venom components from fraction FII.8 using C_18_ column (see Materials and Methods for detail). Peptides were eluted with a linear gradient of solution A (0.12% TFA in water) to 60% solution B (0.1% TFA in acetonitrile) over 60 min (black dashed line). Cm39 was identified in the peak that eluted at 25.2 min as indicated with red arrow. (**B**) Full length amino acid sequence of Cm39 determined by automatic Edman degradation. Cysteine residues are bold.

A number of peptide toxins were isolated from the venom of *C. margaritatus* following a three-step purification scheme. A comprehensive description about the purification and proteomic analysis of these peptides was reported in our earlier publication [29]. The first step was size-exclusion chromatography, resulting in three fractions FI, FII and FIII. Fraction FII, which typically contains the toxic components of the venom, was subjected to ion-exchange chromatography (IEC) using a carboxy-methylcellulose (CMC) column. The resulting 10 subfractions (FII.1-10) were individually separated by HPLC using a C_18_ column and molecular weights (MW) were analyzed for the principal peaks by ESI-MS as shown in the previous publication [29]. A peptide of 3980.2 Da MW was found in sub-fraction FII.8 (Figure 1A) and named “Cm39” after the scorpion *C. margaritatus* and its MW. Cm39 toxin eluted at a retention time (R_T_) of 25.2 min from C_18_ column as indicated in HPLC chromatogram (Figure 1A). The complete sequence of Cm39 was obtained by direct automatic Edman degradation. It has 39 residues with six cysteines and three putative disulfide bridges (Figure 1B).

### 2.2. Chemical Synthesis

**Figure 2:**
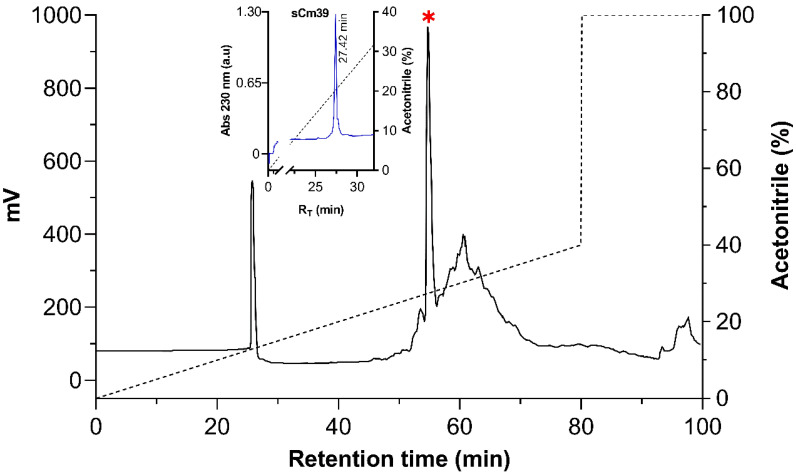
Purification of the synthetic Cm39. After re-folding, sCm39 was purified using C_18_ preparative column (238TP1022 Vydac) with a linear gradient of solution A (0.12% TFA in water) to 40% solution B (0.1% TFA in acetonitrile) over 80 min (black dashed line) and at a flow rate of 5mL/min. Inset shows the re-purification of the main fraction (R_T_ 54.8, indicated with red asterisk) in a C_18_ analytical column (218TP54 Vydac) with a linear gradient from 0 to 60% B solution in 60 min (black dashed line).

In order to obtain a suitable quantity of the Cm39 peptide for functional assay we synthesized it using the solid phase method from Merrifield [31]. The peptide was purified using HPLC, and the main component was analyzed (Figure 2). The MW of synthetic Cm39 (sCm39) obtained was 3980.68 Da and the amino acid sequence determined by automatic Edman degradation showed that the peptide contained the expected sequence.

### 2.3. Pharmacological Properties of Cm39

**Figure 3:**
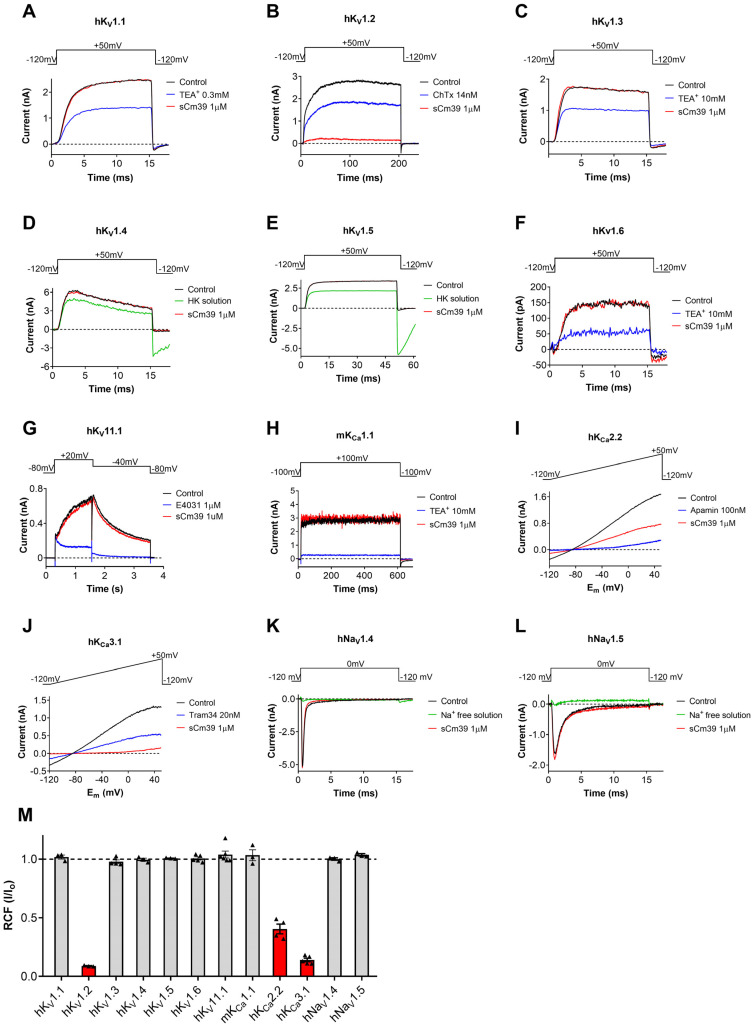
Pharmacological profile of sCm39. (**A**–**L**) Representative current traces displayed in each panel were recorded in the absence of (control trace in black) the toxin and in the presence of 1 µM sCm39 (trace in red) upon reaching the equilibrium block (**B**,**I**,**J**) or after 12−20 depolarization pulses (3−5 min). Solution exchange in the recording chamber was confirmed using fully reversible inhibitors of the channels (traces in blue) or salt solution (traces in green) as positive controls (HK: HK−150 solution with high extracellular (150 mM) K^+^ to reduce the K^+^ driving force or ChTx: charybdotoxin, TEA^+^: tetraethylammonium chloride, Apamin, Tram34 and E4031, known inhibitors of the appropriate channels as indicated). Voltage protocols are displayed above the current traces in each panel. For extracellular and intracellular solution composition see Materials and Methods section. (**M**) Remaining current fraction (RCF, I/I_0_) values were calculated as the ratio of the peak currents in the presence (I) or absence (I_0_) of 1 µM sCm39 at steady-state block (for K_V_1.2, K_Ca_2.2 and K_Ca_3.1) or after 3−5 min of application of toxin. In the case of K_Ca_2.2 and K_Ca_3.1, the peak currents were measured at +48 mV (time point 148 ms) of the ramp. Bars with individual data points (triangles) represent RCF values determined on individual cells, bars in red color highlight ion channels that are potential targets of Cm39. Error bars indicate the mean ± SEM (*n* = 3−5).

The primary sequence of Cm39 has a significant resemblance with several scorpion toxins that are highly potent inhibitors of voltage-gated K^+^ channels. We, therefore, aimed at testing the pharmacological activity of sCm39 on a battery of various potassium and sodium ion channels. Among the tested channels were six members of human voltage-gated K^+^ (hK_V_) channels from Shaker family, hK_V_1.1-hK_V_1.6 (Figure 3A–F) and three members of Ca^2+^-activated potassium channel; hK_Ca_2.2 (SK2, Figure 3I), the small-conductance Ca^2+^-activated channel; hK_Ca_3.1 (IK_Ca_1, SK4. Figure 3J), the intermediate-conductance Ca^2+^-activated channel expressed in T lymphocytes; and mK_Ca_1.1 (BK, Slo1, MaxiK, Figure 3H), the large-conductance voltage- and Ca^2+^-activated channel of mice. In addition, we also screened the effect of Cm39 on hK_V_11.1 (hERG1, Figure 3G), a voltage-gated cardiac K^+^ channel, and two human voltage-gated sodium (hNa_V_) channels, hNa_V_1.4 (Figure 3K) and hNa_V_1.5 (Figure 3L), expressed in skeletal and cardiac muscles, respectively. These ion channels were heterologously expressed in the CHO or HEK cell lines except for K_V_1.3. Human peripheral T lymphocytes. They were stimulated with Phytohemagglutinin A (PHA) to increase the K_V_1.3 channel expression, and Ca^2+^-free intracellular solution was used to avoid K_Ca_3.1 channel activation [30]. Appropriate depolarization protocols were used to record ionic currents in voltage-clamped cells (see Figure 3 insets and Material and Methods for details). Freshly dissolved sCm39 in extracellular solution was applied at 1 µM concentration on the cell using a custom-built micro-perfusion system at ~200 µL/min flow rate. The complete exchange of solution in the recording chamber was ensured by frequently using either fully reversible blockers, such as TEA^+^ for K_V_1.1, K_V_1.3, K_V_1.6 and mK_Ca_1.1; Charybdotoxin (ChTx) for K_V_1.2; and apamin for K_Ca_2.2 or specific solutions that allow testing the solution exchange: HK-150 solution for K_V_1.4 and K_V_1.5; and Na^+^-free solution for Na_V_1.4 and Na_V_1.5 as positive controls. The decrease in the current upon perfusion of the cell with these controls confirmed the nature of ion channel expressed, especially when specific inhibitors were used, and that full solution exchange was achieved.

We found that sCm39 at 1 µM concentration did not exhibit any significant effect on 9 of the 12 tested channels as shown in panels A–L of Figure 3. The summarized selectivity data in Figure 3M indicates that the three channels which were affected by sCm39 were K_V_1.2, K_Ca_2.2 and K_Ca_3.1. At equilibrium block, 1 µM of Cm39 reduced 95% of K_V_1.2, 55% of K_Ca_2.2 and 86% of K_Ca_3.1 currents (Figure 3M) whereas decrease in the current of other channels was <3%. The selectivity profile indicates that sCm39 is selective for K_V_1.2, K_Ca_2.2 and K_Ca_3.1 over the other channels investigated in this study.

### 2.4. Mechanism of K_V_1.2 Block

**Figure 4:**
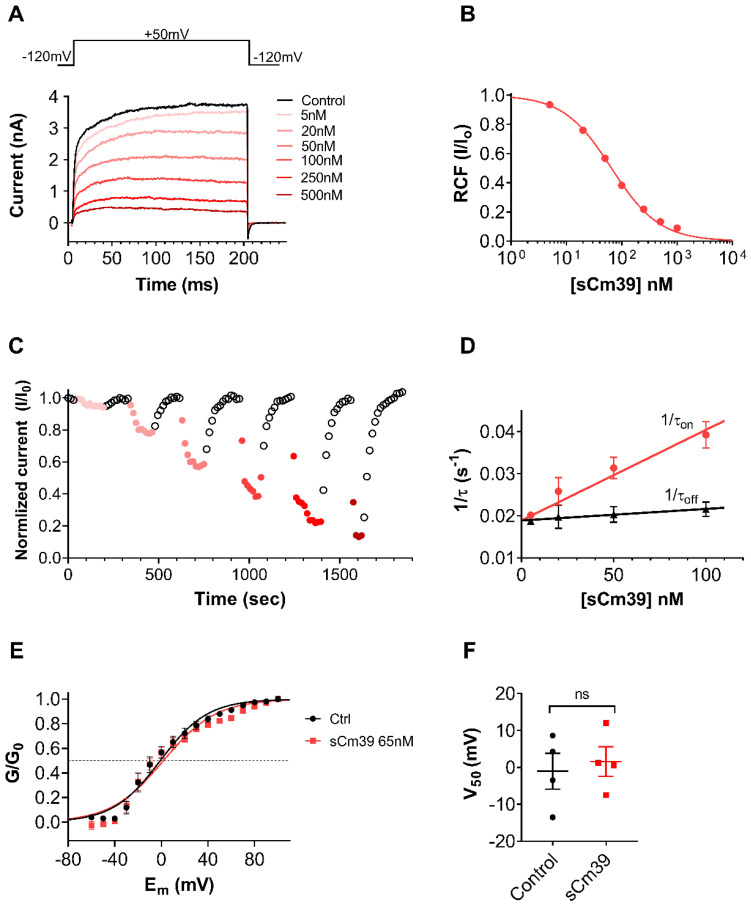
Mechanism of K_V_1.2 block by sCm39. (**A**) The whole-cell K^+^ currents through K_V_1.2 were recorded in transiently transfected CHO cells during 200 ms long depolarization pulses to +50 mV from a V_h_ of −120 mV every 15 s. Representative current traces show the K^+^ current before the application of toxin (control in black) and at equilibrium block upon application of different concentration of sCm39 (5−500 nM, represented in red shades). (**B**) Concentration-dependence of K_V_1.2 current block by sCm39. A Hill equation (see Materials and Methods for details) was fitted to the RCF (I/I_0_) values calculated at different concentrations of sCm39 (solid lines). The best fit yielded a K_d_ of 65 nM and Hill coefficient of 0.96. (**C**) Time course of onset and recovery of the K_V_1.2 current inhibition for the cell shown in panel A. Normalized peak currents were plotted as function of time. Data points in red shades (filled circles) represent the application of sCm39 (5−500 nM) to the bath solution. After attaining the block equilibrium at each concentration of sCM39, cells were perfused with solution lacking the toxin to show reversibility of the block (data points in black, empty circles). (**D**) Effect of sCm39 concentration on the blocking kinetics. The 1/τ_on_ values, (filled circles in red) and dissociation rate constant (1/τ_off_ or k_off,_ triangles in black) were plotted as a function of sCm39 concentration. Data points were fitted with linear regression (r^2^ = 0.97, represented with red and black line for 1/τ_on_ and k_off_, respectively). (**E**) Conductance-voltage (G−V) relationship was constructed by recording the peak K_V_1.2 currents in CHO cells at test potentials ranging from −60 to +100 mV in 10 mV steps from V_h_ of −120 mV. Then, normalized conductance (G/G_0_, with G_0_ being the maximum conductance) was calculated for each test potential using the chord-conductance equation (see Materials and Methods), averaged, and plotted as function of membrane potential (E_m_). Data points were fitted with the Boltzmann sigmoidal equation (solid lines). The G−V relationship was determined in the absence (filled circles in black) or in the presence (filled squares in red) of 65 nM sCm39. The dashed reference line indicates the normalized conductance value of 0.5. (**F**) V_50_ values from G−V relationship of individual cells were determined and plotted. Symbols show individual data points acquired in the absence (filled circles in black) or in the presence (filled squares in red) of 65 nM sCm39. Mann–Whitney test gave *p* = 0.89. ns = not significant. (**C**–**F**) Error bars represent SEM and *n* ≥ 3.

For comprehensive characterization of Cm39 activity on K_V_1.2, we determined the concentration–response of current inhibition by Cm39, the kinetic parameters of its binding and assessed the effect on voltage dependence of steady-state activation. The whole-cell K_V_1.2 currents were recorded in CHO cells using depolarization pulses to +50mV from −120mV holding potential (V_h_) every 15 s. Since the activation kinetics of K_V_1.2 are highly variable [32], the duration of pulses was set on a cell-by-cell basis between 15–300 ms to allocate sufficient time for reaching maximum currents at the test potentials. Figure 4A shows K_V_1.2 current traces recorded sequentially in the same cell in the absence of toxin (control trace) and after perfusing the cell with different concentrations of sCm39 until the equilibrium block. sCm39 reduces the K_V_1.2 currents in a concentration-dependent manner, i.e., 5, 20, 50, 100, 250 and 500 nM inhibited approximately 5, 22, 43, 62, 78 and 87% of current at equilibrium block. The remaining current fractions (RCF = I/I_0_, where I_0_ is the peak current in the absence of the toxin, and I is the peak current at equilibrium block in the presence of sCm39 at a given concentration) were calculated and plotted as a function of toxin concentration. The Hill equation was fit to the data points of the concentration–response relationship (see Materials and Methods), which resulted in a dissociation constant (K_d_) of 65 nM with a Hill coefficient of 0.96 (Figure 4B).

The development and recovery from the block at various concentrations of sCm39 are displayed in Figure 4C, where normalized peak currents were plotted against the time. The block of K_V_1.2 by various concentrations of sCm39 was fully reversible and kinetics of both toxin association and dissociation were very fast. Figure 4D shows the analysis of the kinetic parameters of K_V_1.2 current inhibition by sCm39. Single-exponential decay functions were fitted to the normalized peak currents in the presence of each sCm39 concentration (Figure 4C, data points in red shades) to determine the time constant for the onset of block (τ_on_, wash-in or association time constant). The dissociation time constant for the relief from block (τ_off_, wash-out time constant) was obtained by fitting a single-exponential rising function to the normalized peak currents in the wash-out procedure (Figure 4C, data point in black empty circles). Assuming a simple bimolecular interaction between the toxin and the channel, time constants for the onset and recovery from the block can be stated as follows:

(1) $$\tau_{\mathrm{on}}=\frac{1}{k_{\mathrm{on}}\times \left[\mathrm{toxin}\right]+k_{\mathrm{off}}}\;\;,{\;\tau }_{\mathrm{off}}=\frac{1}{k_{\mathrm{off}}}$$

where k_on_ represents the second-order association rate constant, k_off_ indicates the first-order dissociation rate constant and [toxin] is the sCm39 concentration. The 1/τ_on_ and 1/τ_off_ values were plotted as a function of toxin concentration where 1/τ_on_ rises linearly with the Cm39 concentration; however, the dissociation rate (1/τ_off_) remains constant with a k_off_ value of 0.019±0.0013 s^−1^ (Figure 4D), as also described for the ChTx binding to a Shaker channel [33] and for the sVmKTx interaction with K_V_1.3 [34]. The second-order rate constant of association (k_on_) was determined by fitting the 1/τ_on_ data points using linear regression with k_off_ as the y-intercept. The slope of the regression line corresponds to the k_on_ of $2.15\times 10^{-4}\pm 2.19\times 10^{-5}$ nM^−1^ s^−1^ (r^2^ = 0.97; Figure 4D). The dissociation constants (K_d_ = k_off_/k_on_) calculated from the block kinetic parameters resulted in 87.8 nM, which is comparable to the K_d_ value determined from equilibrium block (Figure 4B).

Mostly, scorpion toxins inhibit the K_V_ channels by physically occluding the pore region of the channel thereby preventing the flow of K^+^ ions. There are some other peptide toxins which bind to the voltage-sensor domains of Kv channels and significantly shift the voltage-dependence of steady-state activation towards depolarized potentials which results in decreased K^+^ current [35,36]. To confirm the blocking mechanism of Cm39 we determined the conductance–voltage (G–V) relationship for K_V_1.2 in the absence and presence of the toxin. Whole-cell currents of K_V_1.2 expressing CHO cells were recorded by applying the 300 ms long depolarization pulses ranging from −70 to +80 in 10 mV steps from V_h_ of −120 mV. Due to the highly variable activation properties of K_V_1.2 [32] we restricted the analysis to the current records which showed similar gating mode. The conductance values were calculated for each voltage step, normalized to the maximum conductance and plotted as a function of membrane potential (E_m_) as shown in Figure 4E. The fitting of averaged data points with the Boltzmann sigmoidal function resulted in the superimposed solid lines (Figure 4E), demonstrating that the presence of 65 nM of sCm9 did not affect the voltage dependence of steady-state activation of K_V_1.2 channels. The midpoint voltage (V_50_) of the G–V relationship in the control solution (−1.0 ± 4.8, *n* = 4) was statistically similar to the V_50_ value at equilibrium block with 65 nM sCm39 (1.6 ± 4.0, *n* = 4) as shown in Figure 4F. These results suggest that Cm39 is not modifying the gating of K_V_1.2, rather it binds to the pore region of the channel.

### 2.5. Cm39 Inhibits Small-Conductance/Intermediate Conductance Ca^2+^-Activated Channels with Nanomolar Affinity

**Figure 5:**
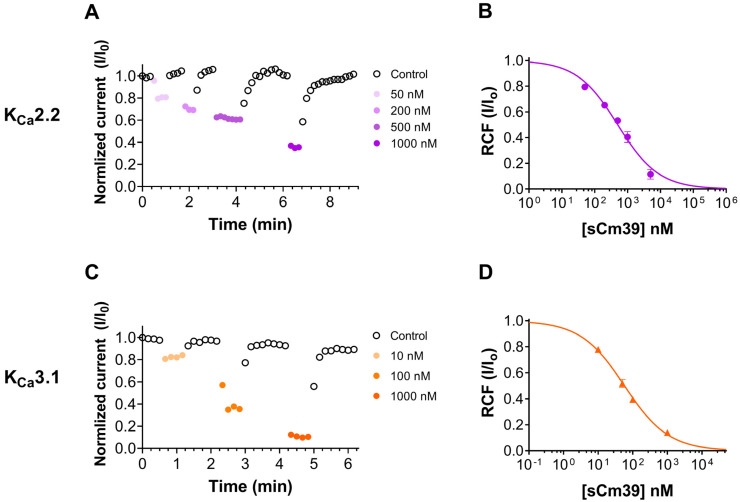
Inhibition of K_Ca_2.2 and K_ca_3.1 currents by sCm39. (**A**,**C**) K^+^ currents of CHO cells expressing K_Ca_2.2 (A) or K_ca_3.1 (**C**) in whole-cell configuration by applying 150 ms long voltage ramp to +50 mV from −120 mV every 10 s. To demonstrate the time course of development and recovery of the K^+^ current inhibition, normalized peak currents (I/I_0_) were plotted as function of time where peak currents were determined at ~+48mV (time point 148 ms) of the ramp. Data points shown with filled circles in purple shades (**A**, K_Ca_2.2) and in orange shades (**C**, K_ca_3.1) represent the application of different concentrations of sCm39 to the bath solution. Following the equilibrium block at each concentration of toxin, cells were perfused with toxin-free solution to exhibit reversibility of the K^+^ current inhibition (data points shown with empty circles in black). (**B**,**D**) Concentration-dependent block of K_Ca_2.2 (**B**) and K_ca_3.1 (**D**) by sCm39. A Hill equation (see Materials and Methods for details) was fitted to the RCF (I/I_0_) values (represented with purple filled circles for K_Ca_2.2 in panel B and with orange triangles for K_Ca_3.1 in panel D) determined at different concentrations of sCm39 (solid lines). The best fit yielded K_d_ = 502 nM, H = 0.7 for K_Ca_2.2 (**B**) and K_d_ = 57.7 nM, H = 0.68 for K_ca_3.1 (**D**). Error bar denotes SEM and *n* ≥ 3.

Cm39 also targets Ca^2+^-activated potassium channels K_Ca_2.2 and K_Ca_3.1 as described in the selectivity profile experiments above; therefore, we studied the effect of Cm39 in concentration-dependent manner on these channels. The whole-cell K^+^ currents of CHO cells transiently expressing K_Ca_2.2 or K_Ca_3.1 channels were recorded sequentially by applying the 150 ms long voltage ramp to +50 mV from V_h_ of −120 mV every 10 s in the presence of control solution or upon perfusing the cell with different concentrations of sCm39. Normalized peak currents were calculated and plotted as function of time as shown in Figure 5A for K_Ca_2.2 and in Figure 5C for K_Ca_3.1 in the presence of the toxin at various concentrations and upon wash-out. Figure 5A,C show that block of both channels by sCm39 was fully reversible, and toxin association and dissociation kinetics were very fast. The development and almost full recovery from the equilibrium block took place in 1–3 depolarization pulses separated by 10 s. RCF values were determined at equilibrium block in the presence of various concentrations of sCm39 and fitted with the Hill equation (see Materials and Methods for details). The resulting dissociation constant (K_d_) values and Hill coefficients (H) were K_d_ = 502 nM, H = 0.7 for K_Ca_2.2 (Figure 5B) and K_d_ = 57.7 nM, H = 0.68 for K_Ca_3.1 (Figure 5D). The affinity of Cm39 for K_Ca_3.1 is ~10 times higher than for K_Ca_2.2.

### 2.6. Comparative Sequence and Phylogenetic Analyses

Amino acid sequence alignment of the different KTx families with Cm39 showed that Cm39 is most closely related to the α-KTx family. Within the α-KTx family, the subfamilies with the highest average identity percentages were: α-KTx 4 with 60.8%, α-KTx 2 with 51.4%, α-KTx 23 with 44.9%, α-KTx 1 with 43.9%, α-KTx 3 and 19 with 43.8%, and α-KTx 12 with 43.5% with the most conserved region being the C-terminus of the peptides. Table 1 shows the alignment between Cm39, the α-KTx 4 subfamily and a representative sequence of the other α-KTxs subfamilies. As can be seen, except for α-KTx 4.2, the other members of subfamily 4 show more than 60% identity to Cm39.

**Figure 6:**
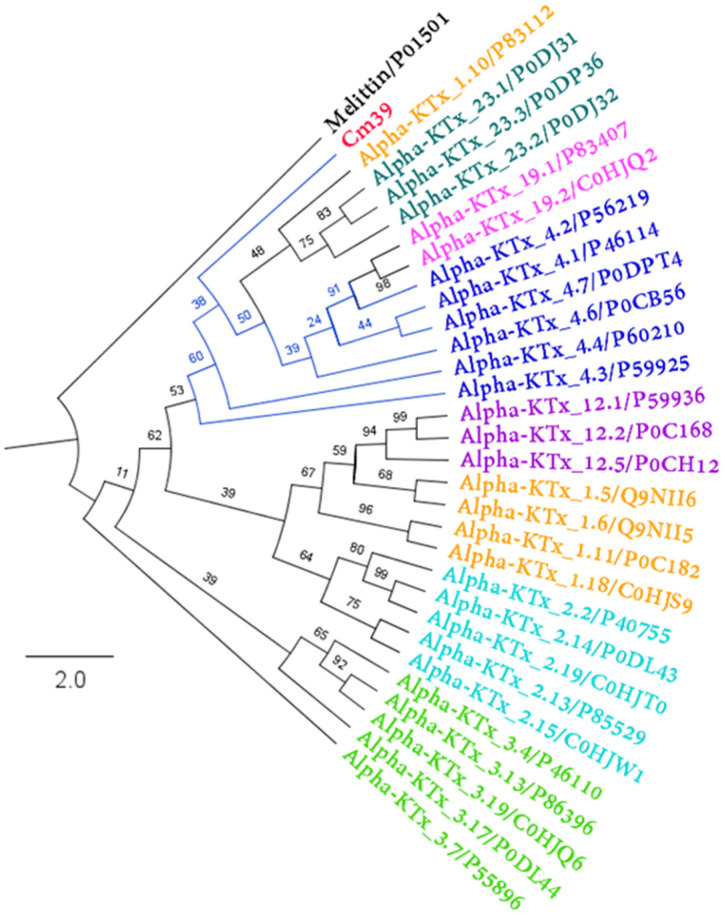
Phylogenetic analysis of Cm39. Maximum likelihood tree topology obtained from the analysis of Cm39 and other related α-KTxs (Log-likelihood = −1284.532). Each color represents a different α-KTx subfamily. Each toxin is indicated with its corresponding UniProt accession number. The numbers in the nodes indicate the bootstrap support values. Melittin protein sequence was used as an outgroup.

Phylogenetic analysis was performed by comparing the amino acid sequence of Cm39 with representative sequences of the most related α-KTxs subfamilies. Cm39 was clustered with all other α-KTx 4 toxins, indicating that Cm39 belongs to this subfamily and that α-KTx 4 toxins are the ancestor of subfamilies 19 and 23 (Figure 6).

## 3. Discussion

In this study, we described the purification, determination of primary structure, chemical synthesis and characterization of pharmacological activities of a new peptide toxin, Cm39, from the venom of *Centruroides margaritatus*. Cm39 consists of 37 residues with six cysteines. In electrophysiological evaluation, it inhibited human K_V_1.2 and intermediate-conductance Ca^2+^-activated (K_Ca_3.1) channel with nanomolar affinity (K_d_ = 65 nM and 58 nM, respectively). Additionally, it also showed a blocking effect on a small-conductance Ca^2+^-activated channel (K_Ca_2.2) with comparatively low affinity (K_d_ = 502 nM). However, Cm39 did not show any effect on several other ion channels tested in this study including five other subtypes of voltage-gated K^+^ channels (K_V_1.1, K_V_1.3, K_V_1.4, K_V_1.5, K_V_1.6 and K_V_11.1), two subtypes of Na_V_ channels (Na_V_1.4 and Na_V_1.5) and the large-conductance Ca^2+^-activated channel (mK_Ca_1.1).

When the amino acid sequence was compared, Cm39 showed high similarity to α-KTxs. Within the α-KTxs family, peptides of subfamily 4 showed a higher percentage of identity with Cm39 (~60%). Most of the peptides in subfamily 4 are very similar (percentage identity ranging from 78 to 97%) and share common pharmacological targets, including K_V_1.3, K_V_1.2 and K_Ca_ channels. Like the subfamily 4 peptides, Cm39 also showed an inhibitory effect on K_V_1.2, K_Ca_2.2 and K_Ca_3.1 channels, but not on the K_V_ 1.3 channel. Not all subfamily 4 toxins have been tested on the K_V_1.3 channel, but for those that were tested (α-KTxs 4.1, 4.4 and 4.6), very similar K_d_ values were found (19.8 nM, 16 nM and 10.7 nM, respectively) [37,38,39]. It has been reported that there are several features in the primary structure of toxins that confer selectivity for the K_V_1.3 or K_V_1.2 channel. A higher overall positive charge, a greater number of basic residues at the C-terminus, a Met residue two positions downstream of the dyad Lys and a residue other than Tyr in the dyad confer selectivity for the K_V_1.3 channel. Contrastingly, a lower overall charge, a lower number of basic amino acids, a Tyr residue in the dyad and an Ile residue two positions downstream of the dyad Lys, confer selectivity for the K_V_1.2 channel [39]. Cm39 fully satisfies some of these characteristics. First, Cm39 has an overall charge of 3.6, which is lower compared to the other toxins mentioned (> 4.8). Second, while the other toxins have two Lys residues at the C-terminus, Cm39 has a change from Lys to Thr (T34). Third, the Met residue in Cm39 has been replaced by Ile (I31), and the Tyr residue is located in the essential dyad. All these together could explain the selectivity of Cm39 towards the K_V_1.2 channel over the K_V_1.3 channel. On the other hand, the effect of subfamily 4 peptides on the K_Ca_2.2 channel has been demonstrated only for the α-KTxs 4.2. The molecular mechanism of interaction with the K_Ca_2 (small-conductance K^+^ channel, SK) was attributed to the conserved motif “RXCQ” found in toxins such as Lei-I and PO5 [40], in addition to apamin, which has a blocking effect in the picomolar range [41]. However, this motif is not present in α-KTxs 4.2 or Cm39, thus the mechanism of interaction is likely to be different. Mutagenesis studies on α-KTx 4.2 have shown that Arg6 and, to a lesser extent, Arg9 are important residues for the interaction of the toxin with the SK channels [42]. In Cm39, Arg6 is replaced by Lys, which also results in a positive charge, and Arg9 is replaced by Ser, although a Lys is present at position 8. These changes could explain the difference in K_d_ value between Cm39 and α-KTxs 4.2 (502 nM and 80 nM, respectively). In addition, these amino acid changes (K6 and K8) are conserved in the other subfamily 4 toxins, and further investigation may help to clarify the molecular mechanism of the interaction of subfamily 4 toxins with the K_Ca_2.2 channel. None of the subfamily 4 toxins have been reported to block the K_Ca_3.1 channel. However, based on sequence similarity with toxin Cm39, it is possible that other members of the α-KTx 4 family also inhibit the K_Ca_3.1 channel. There are two other peptides that have been reported as high affinity blockers of the K_Ca_3.1 channel, charybdotoxin (ChTx, α-KTx-1.1) and maurotoxin (MTX, α-KTx-6.2), whose K_d_ values are 5 nM and 1 nM, respectively [43]. The K_d_ value of these toxins is not far from that found for Cm39 (58 nM); however, none of the toxins is selective for this channel (like Cm39). ChTx shows effects on K_V_1.2 and K_V_1.3 channels [44] and MTX inhibits the Kv1.2 channel [45]. A new toxin such as Cm39 could help to identify the motifs involved in selectivity toward the K_Ca_3.1 channel by expanding the repertoire of peptides with which comparative analysis can be performed to find amino acids likely involved in this interaction. Thus, amino acid sequence and phylogenetic analyses, where clearly Cm39 is clustered within subfamily 4, and the pharmacological properties of Cm39 indicate that it is a novel member of the α-KTx 4 subfamily. Therefore, the Cm39 toxin was registered with the systematic number of α-KTx 4.8, and its primary amino acid sequence will appear in the UniProt Knowledgebase under the accession number C0HM65.

Generally, scorpion toxins interact at two different regions of K_V_ channels to modify their function. Typical functional dyad (lysine and tyrosine) bearing toxins bind to the extracellular vestibule in such a way that lysine goes deep into selectivity filter, thereby plugging the pore of the channel [33,46,47,48]. Other toxins interact with the voltage-sensing domain (VSD) causing the significant change in gating of the channel [35,36]. We found that the voltage dependence of steady-state-activation of K_V_1.2 channel was completely insensitive to the presence of Cm39, thereby clarifying that it does not interfere with the VSD (Figure 4E and F). On the other hand, we propose that Cm39 could bind to the pore region and Lys27 is responsible for obstructing the pore of K_V_1.2 channel. In addition, the binding kinetics analysis showed a simple bimolecular interaction between Cm39 and K_V_1.2 channel as described previously for typical pore blocker toxins such as charybdotoxin and margatoxin [33]. An apparent first-order association rate showed direct correlation with the Cm39 concentration, being faster at higher toxin concentration; however, the first-order dissociation rate was not affected by the change in toxin concentration (Figure 4D). The dissociation constant (K_d_) for K_V_1.2 calculated by the k_off_/k_on_ ratio (87.8 nM) is comparable to the K_d_ value determined by fitting the Hill equation to the concentration dependence of current inhibition (65 nM). Moreover, we observed that the block of all Cm39-sensitive channels (K_V_1.2, K_Ca_2.2 and K_Ca_3.1) was fully reversible and association and dissociation rates of Cm39 were fast. Although Cm39 quickly binds to the channel and blocks the conductance presumably by protruding Lys27 into the selectivity filter, unfavorable interactions between residues of toxin and the external vestibule of the channel destabilizes the toxin binding, thereby drastically shorting the residence time of Cm39 on the channel. Goldstein and Miller have showed this by generating several mutants of ChTx with very high off rates [33]. Similarly, the double substitution in AnTx[N17A/F32T] resulted in unfavorable interaction with the pore of the K_V_1.3 channel and exhibited a significantly fast dissociation rate as compared to the wild-type toxin [49].

To date, a number of scorpion-derived peptide toxins are known to block K^+^ channels with great affinity. However, these native toxins target more than one channel which compromises their therapeutic use in the management of channelopathies [23]. For example, Urotoxin (α-KTx-6.21) [50], MgTx (α-KTx-2.2) [51] and ChTx (α-KTx-1.1) [52] inhibit several K_V_1.x channel subtypes and Ca^2+^-activated channels with high affinity ranging from picomolar to nanomolar. Vm24 (α-KTx-23.1) is a high affinity blocker of the K_V_1.3 channel and its effect is >1500-fold selective for K_V_1.3 over the K_V_1.1, K_V_1.2 and K_Ca_3.1 channels [53]. The selectivity of these potential peptides can be enhanced through protein engineering. For instance, by substituting two residues (N17A/F32T) in Anuroctoxin (α-KTx 6.12) it gained 16,000-fold selectivity for K_V_1.3 over K_V_1.2 while retaining its native affinity for K_V_1.3 [49]. Leiurotoxin I (α-KTx-5.1, Scyllatoxin, isolated from *Leiurus quinquestriatus hebraeus*) is a potent inhibitor of small-conductance Ca^2+^-activated potassium channel K_Ca_2.x subtypes. The Lei-Dab^7^ mutant, a less potent but highly selective blocker for K_Ca_2.2, was designed by replacing single residue with unnatural amino acid diaminobutonoic acid [40]. Recently, our group developed an analog of Vm24 by mutating a single amino acid (K32E) which is still a high affinity blocker of K_V_1.3 while gaining ~9000-fold selectivity over K_V_1.2, while it was insensitive to K_V_1.1 and K_Ca_3.1 at a 2.5 µM concentration [34].

As discussed, Cm39 blocks K_V_1.2 and K_Ca_3.1 with similar affinities and shows comparatively less affinity for the K_Ca_2.2 channel (Figure 4B and Figure 5). Cm39 at a 1µM concentration did not block several other channels included in this study (Figure 3). This pattern of ion channel inhibition influences the potential biological application of Cm39 in its native form. For example, K_V_1.2 is a typical ion channel in the central nervous system which is protected by the blood–brain barrier and thus, restricts the access of peptide blockers to the channels. On the other hand, conjugation of the therapeutic peptides with BBB shuttle peptides is known to increase the applicability of these peptides [54,55] For example, inhibition of K_V_1.2 may have beneficial effects in epilepsy associated with gain of function mutations of K_V_1.2 [14]. Inhibition of K_Ca_3.1, especially in combination with targeting K_Ca_2.2, can be interesting for influencing atrial fibrillation (AF). It was shown recently that inhibition if K_Ca_3.1 or K_Ca_2.2 in isolation may reduce AF [56,57,58]. It may be an interesting scenario to target both ion channels simultaneously, and based on our results Cm39 would be a suitable candidate for a dual-action drug candidate. In addition, selectivity of Cm39 for a particular ion channel over the other channels can be improved through peptide engineering after revealing the candidate residues which interact with the channel vestibule.

## 4. Conclusions

In summary, we characterized a new member of the α-KTx 4 family from the venom of Colombian scorpion *Centruroides margaritatus*. The synthetic Cm39 peptide blocks K_V_1.2 and Ca^2+^-activated potassium channels (K_Ca_2.2 and K_Ca_3.1) with nanomolar affinities while it does not affect several other K^+^ and Na^+^ channels. Cm39 targets two distinct families of potassium channel (voltage-gated and Ca^2+^-activated), and it bears >15-fold selectivity for K_V_1.2 over the K_V_1.3 channel. These properties of Cm39 make it a good candidate for designing a specific inhibitor of a certain potassium channel to use as a therapeutic drug or as a tool for physiological studies.

## 5. Materials and Methods

### 5.1. Isolation and Amino Acid Sequence Determination of Native Cm39

A detailed procedure of venom preparation and purification of peptide toxins including Cm39 (MW 3980 Da) from *C. margaritatus* venom was described previously [29]. Concisely, the soluble venom was first fractionized using the Sephadex G-50 column in 20 mM ammonium acetate buffer of pH 4.7 at a flow rate of 2 mL/min. Then, a toxic peptide containing fraction was subjected to ion exchange chromatography (IEC) using a carboxy-methylcellulose (CMC) column and components were eluted with a linear gradient 0–100% of 500 mM ammonium acetate buffer in 200 min at 2 mL/min flow rate. Lastly, IEC fractions were further purified by high-performance liquid chromatography (HPLC) using analytical C_18_ reverse-phase column (Vydac). To elute the peptides from the column, a linear gradient of 0–60% of solution B (0.1% TFA in acetonitrile) in solution A (0.12% trifluoroacetic acid in water) was run for 60 min at a 1 mL/min flow rate. HPLC fractions were collected manually, and after vacuum drying stored at −20 °C until further analysis. A sample from a single peak of HPLC was analyzed in the LCQ Fleet mass spectrometer coupled with an electrospray ionization (Thermo Fisher Scientific Inc., San Jose, CA, USA) to determine the molecular mass of pure peptide. The amino acid sequence of the peptide was revealed by automated Edman degradation using Biotech PPSQ-31A Protein Sequencer equipment (Shimadzu Scientific Instruments, Inc., Columbia, MD, USA) according to the procedure as described for other components from the same venom [29,30]. First, pure native peptide was loaded directly for sequencing. Then, a reduced and alkylated sample of the same peptide was sequenced to identify the cysteine residues.

### 5.2. Chemical Synthesis and Folding of sCm39

The peptide Cm39 was chemically synthesized by using the Merrifield technique [31]. The synthesis was prepared manually using the standard Fmoc-based solid phase technique on NovaSyn TGA resin (0.25 mmol/g resin). HBTU and HOBT were used as coupling reagents. A three-fold excess of Fmoc amino acids was added during each coupling cycle. The Fmoc group was removed with 20% piperidine in DMF. Unreacted or deblocked free amines were monitored through the ninhydrin test in each cycle of the peptide synthesis. After cleavage and deprotection for 1.5 h at room temperature with reagent K (TFA 84%, phenol 5%, thioanisole 5%, H_2_O 5%, DTT 1%) the crude peptide was precipitated, washed with methyl-tert-butyl-ether, then dissolved in 20% acetic acid and lyophilized. The cyclization reaction to form disulfide bridges was carried out in 0.1 M NaCl, 5 mM GHS, 0.5 mM GSSG, 20 mM Na_2_HPO_4_ (pH 7.9 with 1M NaOH) at 0.1mg/mL of crude peptide for 2 h then the pH was adjusted to 3 with formic acid. The cyclized peptide was purified in a C_18_ reverse-phase HPLC preparative column (238TP1022 Vydac) with a linear gradient of solution A (0.12% TFA in water) to 40% solution B (0.1% TFA in acetonitrile) over 80 min at a flow rate of 5ml/min. The principal component was re-purified in a C_18_ analytical column (218TP54 Vydac) with a linear gradient from 0 to 60% B in 60 min.

### 5.3. Electrophysiology

#### 5.3.1. Cell Culture

Human peripheral blood monocytes (PBMCs) were isolated from anonymous healthy donors’ blood through Histopaque1077 (Sigma-Aldrich) separation technique and cultured in Roswell Park Memorial Institute (RPMI) 1640 medium (Gibco, Grand Island, NY, USA) containing 2 mM L-glutamine, 10% fetal bovine serum (FBS, Sigma-Aldrich), 100 µg/mL streptomycin and 100 U/mL penicillin-g at a density of $5\times 10^{5}$ cells per mL in a humidified incubator at 37 °C and 5% CO_2_. Phytohemagglutinin A (Sigma-Aldrich) was also added to the medium at a concentration of 2, 5, 10 µg/mL to upregulate the expression of K^+^ channels in PBMCs. Patch-clamp experiments were performed after 3–6 days of activation.

Chinese hamster ovary (CHO) cells (gift from Yosef Yarden, Weizmann Institute of Science, Rehovot, Israel) were maintained by culturing in Dulbecco’s modified Eagle medium (DMEM, Gibco) supplemented with 2 mM L-glutamine, 10% FBS, 100 µg/mL streptomycin and 100 U/mL penicillin-g (Sigma-Aldrich) at a density of 0.$5-1\times 10^{6}$ cells per mL in a humidified incubator at 37 °C and 5% CO_2_. Cells were passaged 3 times in a week following a 2–5 min incubation in 0.05% trypsin-EDTA solution at 37 °C. Cultures were used up to passage number 20. PCR-based tests were routinely used to detect mycoplasma infection, only mycoplasma-free cultures were used for experiments.

#### 5.3.2. Heterologous Expression of Ion Channel

CHO cells that do not show endogenous voltage-gated ion currents [59] were transiently transfected with the following ion channel coding plasmids: hK_V_1.1 (*KCNA1* gene), hK_V_1.2 (*KCNA2* gene) and hK_V_1.6 (*KCNA6* gene) in a pCMV6-AC-GFP plasmid (OriGene Technologies), hK_V_1.4 (*KCNA4* gene) in a pCDNA3 plasmid, hK_V_1.5 (*KCNA5* gene) in a pEYFP-C1 plasmid (a kind gift from A. Felipe, University of Barcelona, Barcelona, Spain), hK_Ca_2.2 (*KCNN2* gene) in a pCDN3 plasmid (a kind gift from Bernard Attali, Tel Aviv University, Israel) hK_Ca_3.1 (*KCNN4* gene) in a pEGFP-C1 vector (a kind gift from H. Wulff, University of California, Davis, CA) and hNa_V_1.5 (*SCN5A* gene, a kind gift from H. Abriel, University of Bern, Bern, Switzerland). Those plasmids which lack a fluorescent tag were co-transfected with a plasmid encoding GFP. Transfections were performed at a GFP:channel DNA molar ratio of 1:10 using the Lipofectamine 2000 kit (Invitrogen) according to the manufacturer’s protocol. GFP expressing cells were identified with the Nikon TE 2000U fluorescence microscope (Nikon, Tokyo, Japan) and used for current recordings (~60–70% success rate for co-transfection). Generally, currents were recorded 24 to 48 h post transfection.

Human embryonic kidney (HEK) 293 cells stably expressing mK_Ca_1.1(BK_Ca_, *Kcnma1* gene, a kind gift from C. Beeton, Baylor College of Medicine, Houston, TX, USA), hK_V_11.1 (*hERG1*, *KCNH2* gene, a kind gift from H. Wulff, University of California, Davis, CA, USA) or hNa_V_1.4 (*SCN4A* gene, a kind gift from P. Lukács, Eötvös Loránd University, Budapest, Hungary) were used.

#### 5.3.3. Solutions

For the measurement of K_V_1.x, mK_Ca_1.1 and Na_V_1.x currents the extracellular solution was composed of (in mM) 145 NaCl, 5 KCl, 2.5 CaCl_2_, 1 MgCl_2_, 10 HEPES and 5.5 glucose, pH 7.35. In the HK-150 and Na^+^-free bath solution bath all Na^+^ was substituted by K^+^ or Choline-Cl, respectively, other ingredients remained unchanged. In the various TEA^+^-containing solutions, Na^+^ was substituted by tetraethylammonium-Cl in equimolar concentration (Figure 3). The bath solution for K_Ca_2.2 and K_Ca_3.1 consisted of (in mM) 145 L-Aspartic Na^+^ salt, 5 KCl, 2.5 CaCl_2_, 1 MgCl_2_, 5.5 glucose and 10 HEPES, pH 7.4 and for K_V_11.1 (in mM) 140 Choline-Cl, 5 KCl, 2 MgCl_2_, 2 CaCl_2_, 10 HEPES, 20 glucose and 0.1 CdCl_2_, pH 7.35. The osmolarity of bath solutions was ranging between 302 to 308 mOsm/L. A total of 0.1 mg/mL bovine serum albumin (BSA, Sigma-Aldrich, Budapest, Hungary) was added to all the bath solutions before the patch-clamp assay to avoid toxin adsorption to the plastic surfaces of the perfusion system. Internal or pipette solution for recording K_V_1.x and mK_Ca_1.1 currents consisted of (in mM) 140 KF, 2 MgCl_2_, 1 CaCl_2_, 11 EGTA and 10 HEPES, pH 7.22. The composition of the internal solution for sodium channels was (in mM) 10 NaCl, 105 CsF, 10 HEPES and 10 EGTA, pH 7.2; for K_V_11.1 (in mM) 140 KCl, 2 MgCl_2_, 10 HEPES and 10 EGTA, pH 7.3; and for K_Ca_ channels (in mM) 150 K-Aspartate, 5 HEPES, 8.5 CaCl_2_, 2 MgCl_2_ and 10 EGTA, pH 7.22. The osmolarity of internal solutions was ~ 295 mOsm/L.

#### 5.3.4. Patch-Clamp Recording Conditions

Whole-cell currents were recorded in voltage-clamped cells following standard protocols [60] by using a Multiclamp 700B amplifier connected to a computer with Axon Digidata1440 digitizer and for data acquisition, Clampex 10.7 software was used (Molecular Devices). Micropipettes were pulled from GC150F-7.5 borosilicate capillaries (Harvard Apparatus) resulting in electrodes having 3–5 MΩ resistance in the bath solution. Current traces were lowpass-filtered through the built-in analog 4-pole Bessel filters of the amplifiers and sampling frequency was set at 4–50 kHz, at least twice the filter cutoff frequency. Current records were discarded when the leak current at V_h_ was >10% of peak current at the depolarization potential. Recordings were carried out at room temperature (20–25 °C). The cell was perfused with control and test solutions by using a gravity-flow perfusion system and excess bath solution was removed constantly by vacuum suction. Voltage protocols were not corrected for changes in the liquid–junction potentials (typically < 5 mV) upon perfusion of the recording chamber with different K^+^ or Na^+^ concentration solutions (see above), as these controls were merely used to confirm solution exchange.

In general, the V_h_ was set at −120 mV and the depolarization pulses were applied every 15 s except when indicated. For recording the K^+^ currents from K_V_1.x, 15–300 ms long voltage pulses to +50 mV were applied. To record the K_V_1.2 currents for the G–V relationship, CHO cells were depolarized to voltages ranging from –60mV to +100 mV in steps of 10 mV every 15 s. For K_V_11.1 channels, currents were evoked with voltage steps to +20 mV for 1.25 s from a V_h_ of –80 mV followed by a step to –40 mV for 2 s, during which peak currents were recorded and pulses were delivered every 30 s. mK_Ca_1.1 currents were recorded by applying voltage steps to +100 mV for 600 ms from a V_h_ of −100 mV. To record the K_Ca_2.2 and K_Ca_3.1 channel currents, a 150 ms long voltage ramp to +50 mV from −120 mV was applied every 10 s. Na^+^ currents through Na_V_1.x were recorded with 15 ms long voltage steps to 0 mV every 10 s.

#### 5.3.5. Patch-Clamp Data Analyses

Patch-clamp data was analyzed using the pClamp 10.7 software package (Molecular Devices). Generally, before analysis current records were corrected for ohmic leakage and digitally filtered with 3-point boxcar smoothing. Each data point in concentration-dependent dose curves represents the mean of ≥3 individual records and these data points were fitted with the Hill equation:

(2) $$\mathrm{RCF}=\frac{K_{d}^{H}}{K_{d}^{H}+{\left[\mathrm{Tx}\right]}^{H}}$$

where RCF is the remaining current fraction (RCF = I/I_0_, where I_0_ is the peak current in the absence of the toxin, and I is the peak current at equilibrium block at a given concentration of toxin), [Tx] is the concentration of the toxin, K_d_ is the dissociation constant and H is the Hill coefficient. When the voltage ramp protocol was used to record the Ca^2+^-activated K^+^ currents, the peak currents were measured at +48 mV (time point 148 ms) of the ramp. Moreover, in the case of K_Ca_2.2 the RCF values at more than 1µM concentration of Cm39 were normalized to the RCF value obtained at 100 nM of Apamin (Smartox Biotechnology, France) to eliminate the influence of any contaminating current. To determine the voltage dependance of steady-state activation of K_V_1.2 current, peak conductance (G) for each test potential was calculated from peak current (I_0_) at a test potential (E_m_) and K^+^ reversal potential (E_K_) by using the chord-conductance equation $G=I_{0}/\left(E_{m}-E_{K}\right)$. The G values were normalized to the maximum value and plotted as a function of test potential and data points were fitted with Boltzmann sigmoidal equation:

(3) $$G_{\mathrm{norm}}=\frac{1}{1+e^{\left(\frac{V_{50}-V}{k}\right)}}$$

where G_norm_ is the normalized conductance, V is the test potential, V_50_ is the midpoint voltage and k is the slope factor of the function.

To study the Cm39 binding kinetics, the association and dissociation rate constants (k_on_, k_off_) for the K_V_1.2 channel were determined following the procedure as described previously [30,34]. Normalized peak currents (I_norm_ = I_t_/I_0_, where I_t_ is peak current in the presence of the toxin at time t and I_0_ is peak current in the absence of toxin) were plotted against the time and data points during the wash-in and wash-out procedures and were fitted with single-exponential function as shown below, to determine the time constants for association (τ_on_) and dissociation (τ_off_) of toxin.

(4) $$I_{\mathrm{norm}}\left(t\right)=\mathrm{RCF}+\left(\left(1-\mathrm{RCF}\right)\times e^{-\frac{t}{\tau }}\right)$$

Then, k_on_ and k_off_ values were calculated from these time constants assuming a simple bimolecular interaction between the channel and toxin, and by using equations shown below, also demonstrated previously in detail [30,33]:

(5) $$k_{\mathrm{on}}=\frac{1-\left(\tau_{\mathrm{on}}\times k_{\mathrm{off}}\right)}{\tau_{\mathrm{on}}\times \left[\mathrm{toxin}\right]},{\;k}_{\mathrm{off}}=\frac{1}{\tau_{\mathrm{off}}}\;$$

#### 5.3.6. Statistics

For statistical analyses and graph plotting, GraphPad Prism software (version 8.0.1) was used. Data points were always given as mean ± SEM. Student’s *t* test with Mann–Whitney rank sum test was performed for pairwise comparison.

### 5.4. Comparative Sequence Analysis of Cm39 with Other Scorpion Toxins

Scorpion toxins belonging to all KTxs families (α, β, δ, γ, λ, ε, and κ) were retrieved from KALIUM (Database of polypeptide ligands of potassium channels) [23]. All amino acid sequence alignments were performed using mafft v7.475 [61]. Phylogenetic analysis by maximum likelihood was performed using iqtree v2.2.0 [62]. Iterative maximum likelihood analyses were performed with the KTxs sequences most related to Cm39. The best substitution model was determined using ModelFinder [63]. Phylogenetic analysis of Cm39 was determined using the PMB+G4 model with 10,000 ultrafast bootstraps [64]. The tree was edited using FigTree1.4.4.

## Figures and Tables

**Table 1:** Alignment of Cm39 and α-KTxs amino acid sequences.

Peptide	Sequence	%ID	Access Number
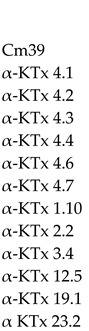	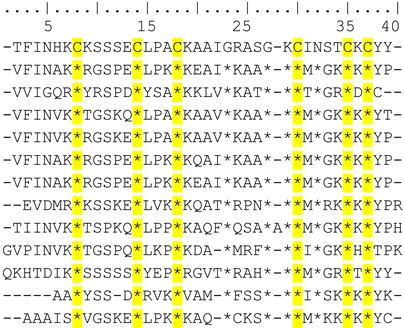	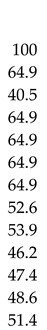	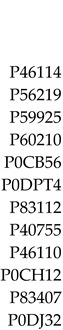

%ID, Percent amino acid identity. The Access number shows the code to access the UniProt database. Conserved cysteine residues are highlighted in yellow. An asterisk (*) indicates identical positions to those in Cm39.

## Data Availability

The raw data supporting the conclusions of this article will be made available by the authors, without undue reservation.
